# Persistence of biologic treatments in psoriatic arthritis: a population-based study in Sweden

**DOI:** 10.1093/rap/rkaa070

**Published:** 2020-12-19

**Authors:** Kirk Geale, Ingrid Lindberg, Emma C Paulsson, E Christina M Wennerström, Anna Tjärnlund, Wim Noel, Dana Enkusson, Elke Theander

**Affiliations:** 1 Quantify Research, Stockholm, Sweden; 2 Department of Public Health and Clinical Medicine, Umeå University, Umeå, Sweden; 3 Janssen-Cilag AB, Solna, Sweden; 4 Department of Epidemiology Research, Statens Serum Institut, Copenhagen, Denmark; 5 Janssen Pharmaceutica NV, Beerse, Belgium

**Keywords:** psoriatic arthritis, biologic therapy, anti-rheumatic agents, autoimmune diseases

## Abstract

**Objectives:**

TNF inhibitors (TNFis) and IL inhibitors are effective treatments for PsA. Treatment non-persistence (drug survival, discontinuation) is a measure of effectiveness, tolerability and patient satisfaction or preferences in real-world clinical practice. Persistence on these treatments is not well understood in European PsA populations. The aim of this study was to compare time to non-persistence for either ustekinumab (IL-12/23 inhibitor) or secukinumab (IL-17 inhibitor) to a reference group of adalimumab (TNFi) treatment exposures in PsA patients and identify risk factors for non-persistence.

**Methods:**

A total of 4649 exposures of adalimumab, ustekinumab, and secukinumab in 3918 PsA patients were identified in Swedish longitudinal population-based registry data. Kaplan–Meier curves were constructed to measure treatment-specific real-world risk of non-persistence and adjusted Cox proportional hazards models were estimated to identify risk factors associated with non-persistence.

**Results:**

Ustekinumab was associated with a lower risk of non-persistence relative to adalimumab in biologic-naïve [hazard ratio (HR) 0.48 (95% CI 0.33, 0.69)] and biologic-experienced patients [HR 0.65 (95% CI 0.56, 0.76)], while secukinumab was associated with a lower risk in biologic-naïve patients [HR 0.65 (95% CI 0.49, 0.86)] but a higher risk of non-persistence in biologic-experienced patients [HR 1.20 (95% CI 1.03, 1.40)]. Biologic non-persistence was also associated with female sex, axial involvement, recent disease onset, biologic treatment experience and no psoriasis.

**Conclusion:**

Ustekinumab exhibits a favourable treatment persistency profile relative to adalimumab overall and across lines of treatment. The performance of secukinumab is dependent on biologic experience. Persistence and risk factors for non-persistence should be accounted for when determining an optimal treatment plan for patients.

**Rheumatology key messages**

- Ustekinumab is associated with a favourable persistence profile compared with adalimumab regardless of biologic experience.
- Secukinumab is associated with a favourable persistence relative to adalimumab in biologic-naïve patients but not in biologic-experienced patients.
- Those with risk factors associated with low persistence should be identified to improve patient outcomes.

## Introduction

PsA is a chronic, heterogeneous, immune-mediated seronegative arthritis characterized by joint inflammation, usually in people with psoriasis, that is estimated to have a prevalence of 0.05–0.42% in the general population in Europe [1] and 6–42% in the psoriasis population [2–4], and specifically 30% in Sweden [5]. The disease is associated with substantial impact on quality of life and economic burden [2].

In recent years, several effective biologic treatments, including TNF inhibitors (TNFis), IL-12 and IL-23 inhibitors and IL-17 inhibitors have been approved for the treatment of PsA. These are highly effective but relatively expensive treatments for PsA. Treatment persistence (also called drug survival or retention), defined as the time from treatment initiation to discontinuation [6], occurs until patients do not continue dispensing treatment or switch to a replacement therapy. Non-persistence is an important real-world endpoint, as it may be viewed as a composite of treatment effectiveness, safety, tolerability and patient satisfaction or preference in the real world [7].

In order to improve patient outcomes and efficiently allocate healthcare resources, the risk factors associated with biologic non-persistence need to be understood in contemporary clinical practice. In addition to the treatments themselves, previous research indicates that prior biologic treatment experience, time from PsA onset to treatment initiation, higher BMI, higher disease activity, age, female sex and the presence of comorbidities, among other factors, are associated with a higher risk of biologic non-persistence [8–20]. Risk factors may differ between those patients with and without biologic experience [8], and persistence rates have been found to be higher in biologic-naïve patients [8–10, 17, 19].

Several studies have examined biologic persistence in PsA patients, most commonly of TNFi therapies [8–21], and recent work has studied persistence in IL-12/23 and IL-17 inhibitors in PsA [22–25]. None of these studies examined European populations and none included both ustekinumab (UST) and secukinumab (SEC).

We conducted an observational, retrospective study of persistence in a Swedish PsA cohort using specialist population-based registry data with lifetime follow-up. The objective was to compare the time to non-persistence for IL-12/23 inhibitor [ustekinumab; Anatomical Therapeutic Chemical (ATC) L04AC05] and IL-17 inhibitor (secukinumab; ATC L04AC10) compared with a reference group of TNFi [adalimumab (ADA); ATC L04AB04] treatment exposures. The association between the non-persistence rate and other risk factors, including biologic treatment experience, was also assessed.

## Methods

### Data and ethics

Population-based national health data from three administrative registries in Sweden, including the National Patient Registry (NPR), Prescribed Drug Registry (PDR) and Cause of Death Registry (CDR), were extracted for use in the present study. Patient-level data from each registry was linked using a unique personal identification number. The NPR includes International Classification of Disease, Tenth Revision (ICD-10) diagnosis codes and corresponding contact dates at each in- and outpatient visit to specialist care providers. The PDR includes data on all pharmacy-dispensed medications, including ATC codes and dispensation dates, from prescriptions originating in primary or specialist care. The CDR includes the patient’s date of death. The study was approved in January 2018 by the Stockholm Regional Ethical Review Board (reference number 2017/2500-31).

### Study population

Patients were included in the study if they had a PsA diagnosis (ICD-10 code L40.5) in the primary position during admission recorded in the NPR between 1 January 2001 and 31 December 2017 and a dispensation of ADA, UST or SEC in the PDR between 1 January 2008 and 30 September 2018. The unit of analysis was treatment exposure, beginning at the initiation of ADA, UST or SEC (index date). Patients could have multiple treatment exposures. Patients were excluded if they had been dispensed ADA, UST or SEC before PsA onset; had been dispensed ADA, UST or SEC before it was approved for PsA indication in Sweden; if they were <18 years of age at the index date or if they did not have a region of residence recorded in the PDR at the exposure index date.

### Non-persistence

Non-persistence was defined as a composite endpoint consisting of treatment switch to any PsA-indicated biologic or Janus kinase (JAK) inhibitor different from the current regimen [including abatacept (ATC L04AA24), adalimumab (ATC L04AB04), certolizumab pegol (ATC L04AB05), etanercept (ATC L04AB01), golimumab (ATC L04AB06), infliximab (ATC L04AB02), ixekizumab (ATC L04AC13), secukinumab (ATC L04AC10), ustekinumab (ATC L04AC05), tofacitinib (ATC L04AA29)] or failure to redispense ADA, UST or SEC within a reasonable time frame (the ‘grace period’) following consumption of all supplied medication. The grace period, defined as the number of days between the end of drug supply and redispensation during which a patient is considered to be on active treatment, was set dynamically to the number of days of drug supplied in the primary analysis. As a sensitivity analysis, a fixed 90 day grace period in addition to the number of days of drug supplied was used for all treatments. Tofacitinib, a PsA-indicated JAK inhibitor, was also included in the study, but we use ‘biologic’ experience throughout this work to refer to both biologic and JAK inhibitors, as fewer than three patients in the present study were treated with tofacitinib.

Drug supplied was calculated as total milligrams dispensed divided by maintenance dose posology, as the administrative registry data used in this study contain the volume of drug dispensed but not dosing, weight or consumption information. As per the Summary of Product Characteristics (SmPC), it was assumed that UST patients’ weight corresponded to the amount of drug dispensed (i.e. ustekinumab dispensations, whether 45 mg or 90 mg vials, always provide 12 weeks of supply before the next administration is required) [26]. Furthermore, as per the SmPC, SEC patients with prior TNFi experience were assumed to consume 300 mg/month while all others consumed 150 mg/month [27]. Adalimumab patients were assumed to consume 40 mg every 2 weeks [28].

### Study design

**Fig. 1:**
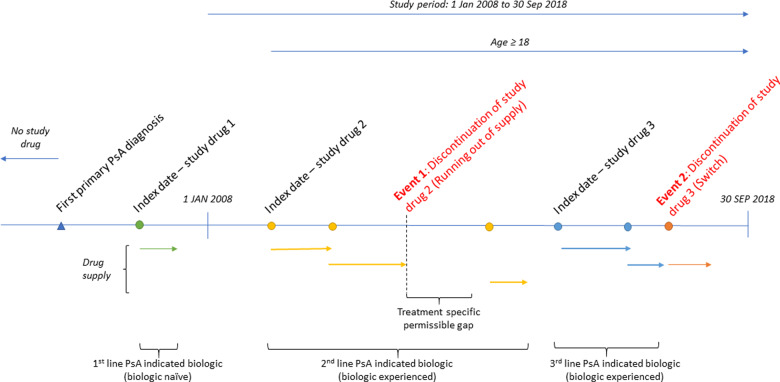
Schematic of the study design Patients initiating ADA, UST or SEC between 1 January 2008 and 30 September 2018 were included in the study. The unit of analysis was treatment exposure, so a single patient could be included in each of the three treatment groups. Non-persistence was defined as a switch to a different PsA-indicated treatment regimen or failure to redispense the same treatment within a reasonable time frame (grace period). Treatment exposures of ADA, UST and SEC were classified as biologic naïve or biologic experienced.

Patients were followed retrospectively and time at risk of non-persistence was defined as treatment initiation (index) until a non-persistence event or censoring [death, end of data (30 September 2018) or 31 December 2015 for Skåne patients (from 2016 and onwards, UST was administered at hospitals in the Skåne region and thus was not included in the PDR and therefore no treatment exposures of ADA, UST or SEC were included in 1 January 2016 onwards for patients living in Skåne)] (**Fig. 1**). A complete-case analysis was applied to the population. Imputation was conducted, where possible, and minimal instances of missing data were subsequently dropped.

### Statistical analysis

Time to non-persistence was visualized using Kaplan–Meier curves and analysed using a Cox proportional hazards model. Unadjusted hazard ratios (HRs) were reported in addition to HRs adjusted for age, time since disease onset, index year, sex, marital status, PsA-indicated biologic treatment experience and comorbidity [represented by the Charlson Comorbidity Index (CCI)] [29]. The presence of ICD-10 diagnosis codes for psoriasis (L40.0–4, L40.8–9), Crohn’s disease (K50+), rheumatoid arthritis (M05+, M06+), axial involvement (M45+, M47+), ulcerative colitis (K51+), uveitis (H20+) and type 2 diabetes (E11+) were also included as covariates. Adjustments were also made for year of index date and region of residence. The CCI and other diagnosis codes were derived from in- and outpatient specialist care data and assessed during the 2 years prior to and including the index date (see Supplementary Tables S1 and S2, available at *Rheumatology Advances in Practice* online). Time since disease onset was defined as the number of years between the first observed PsA diagnosis in NPR during 2001–2017 (PsA disease onset) and biologic treatment initiation (index date). The proportionality assumption of the Cox model was visually inspected and tested using Schoenfeld residuals.

Data management and statistical analyses were conducted in Stata version 15.1 (StataCorp, College Station, TX, USA) and graphics were produced in R version 3.5.1 (R Foundation for Statistical Computing, Vienna, Austria) using ggplot2 [30]. A two-sided type I error (α) of 0.05 was used for all statistical tests.

## Results

A total of 4649 treatment exposures across 3918 PsA patients were included in the study: 3255 to ADA, 507 to UST and 887 to SEC. The maximum actual follow-up duration was 10.6, 5.0 and 2.8 years for ADA, UST and SEC exposures, respectively, depending on each treatment’s PsA approval date in Sweden (August 2005 for ADA, September 2013 for UST and November 2015 for SEC) [31].

In the study population, relatively more SEC exposures were female, more UST exposures had a recorded psoriasis diagnosis and IL inhibitor exposures were initiated later from disease onset and more often biologic experienced (Table 1).

**Table 1:** Summary of patient characteristics

Characteristics	Adalimumab (*n* = 3255)		Ustekinumab (*n* = 507)		Secukinumab (*n* = 887)		*P*-value^b^ (drug cohorts)
	Mean	s.d.	Mean	s.d.	Mean	s.d.	
Age, years	50	13	51	12	52	12	<0.01
Time since disease onset, years	5.0	4.1	7.3	4.7	7.9	4.9	<0.01
CCI score	0.2	0.5	0.2	0.5	0.2	0.6	0.63

Prior record of immunological diagnosis in specialist care^a^	*n*	%	*n*	%	*n*	%	
Psoriasis (L40.0-4, L40.8-9)	1193	37	275	54	337	38	<0.01
RA (M05+, M06+)	310	10	37	7	51	6	<0.01
Crohn’s disease (K50+)	89	3	10	2	<10	<1	<0.01
Ulcerative colitis (K51+)	74	2	<10	<2	12	1	0.17
Axial involvement (M45+, M47+)	123	4	<10	<2	32	4	0.04
Uveitis (H20+)	90	3	<10	<2	15	2	0.11
Type 2 diabetes (E11+)	134	4	38	7	54	6	<0.01
Female	1707	52	281	55	533	60	<0.01
Married	1538	47	223	44	440	50	0.13
Biologic treatment naïve	1990	61	73	14	114	13	<0.01
Biologic treatment experienced	1265	39	434	86	773	87	<0.01

a Derived using ICD-10 diagnosis codes (see Supplementary Table S1 and S2).

b *P*-values for continuous variables were assessed using analysis of variance and chi-square tests were performed for categorical variables.

**Fig. 2:**
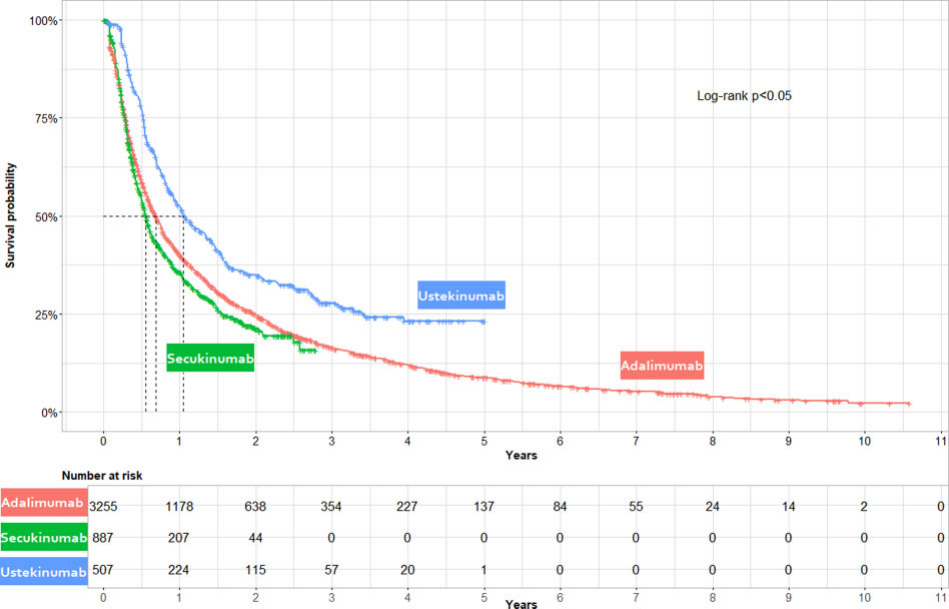
Unadjusted Kaplan–Meier curves of time to non-persistence stratified by treatment Kaplan–Meier curves illustrating unadjusted time to non-persistence by study drug cohort (ADA, UST and SEC). Non-persistence was a composite measure of treatment switch to any other PsA-indicated biologic or failure to redispense ADA, UST or SEC within two times the days supplied following consumption of all supplied medication. Patients were censored at death or the end of available data. Non-persistence rates were highest for SEC exposures, followed by ADA and UST. A log-rank test showed a statistically significant difference between the three curves.

In the unadjusted model, UST had a lower risk of non-persistence relative to ADA [HR 0.67, (95% CI 0.60, 0.75)] while SEC had a higher risk [HR 1.12 (95% CI 1.03, 1.23)] (see Supplementary Table S3, available at *Rheumatology Advances in Practice* online). These findings were corroborated in the unadjusted Kaplan–Meier curves (Fig. 2), where the median survival was 0.55, 0.68 and 1.05 years for SEC, ADA, and UST, respectively. A log-rank test showed that the ADA, SEC and UST non-persistence rates were significantly different from each other (*P* < 0.05).

**Fig. 3:**
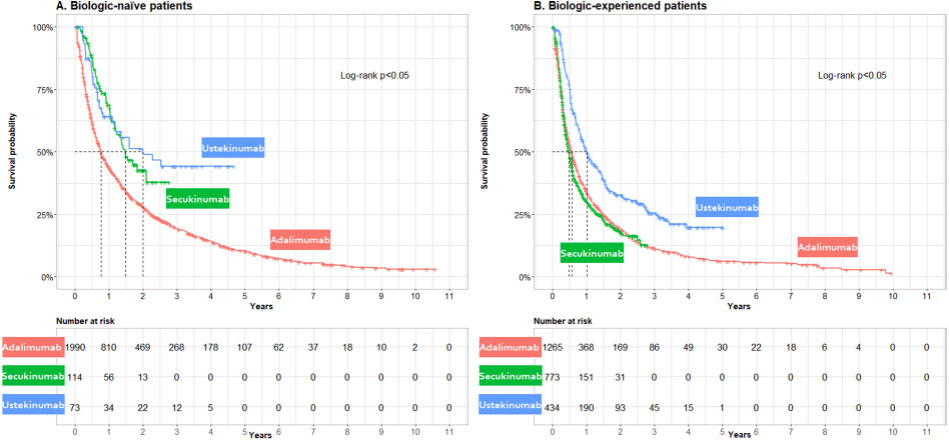
Unadjusted Kaplan–Meier curves of time to non-persistence stratified by treatment and biologic treatment experience Non-persistence was a composite measure of treatment switch to any other PsA-indicated biologic or failure to redispense ADA, UST or SEC within two times the days supplied following consumption of all supplied medication. Patients were censored at death or the end of available data. Log-rank test *P*-values were presented in each panel. (**A**) The median non-persistence in the biologic-naïve cohort was highest for ADA, follwed by SEC and UST. (**B**) The median non-persistence in the experienced cohort was highest for SEC, followed by ADA and UST.

Kaplan–Meier curves were also constructed within biologic treatment experience stratifications (Fig. 3). The median survival (years) was longer in biologic-naïve *vs* experienced ADA exposures (0.77 *vs* 0.56), UST exposures (2.00 *vs* 1.02) and SEC exposures (1.48 *vs* 0.49).

In the adjusted Cox models, UST exposures in the overall, biologic-naïve and biologic-experienced populations had a significantly lower risk of non-persistence than ADA. Conversely, SEC exposures had significantly lower risk of non-persistence in biologic-naïve patients but a higher risk of non-persistence in the biologic-experienced group (see Table 2). As a general trend, the risk of non-persistence appeared to be increasing with biologic experience. Women had a higher risk of non-persistence, as did those initiating treatment closer to disease onset. Those with a diagnosis of psoriasis in specialist care at baseline were associated with lower non-persistence rates overall and in biologic-experienced patients, and those with a specialist care diagnosis of axial involvement at baseline were associated with a shorter time to non-persistence in the overall group. See Supplementary Table S4, available at *Rheumatology Advances in Practice* online, for adjustment for additional covariates, including county of residence and index year. A test of proportional hazards was not rejected in the overall, biologic-naïve or biologic-experienced Cox regressions.

**Table 2:** Results from adjusted Cox proportional hazards model: time to treatment non-persistence

Variables	Overall (*n* = 4649)			Biologic naïve (*n* = 2177)			Biologic experienced (*n* = 2472)		
	HR	95% CI	*P*-value	HR	95% CI	*P*-value	HR	95% CI	*P*-value
Treatment (*vs* adalimumab)									
Ustekinumab	0.56	0.49, 0.64	<0.01	0.48	0.33, 0.69	<0.01	0.65	0.56, 0.76	<0.01
Secukinumab	1.01	0.88, 1.15	0.91	0.65	0.49, 0.86	<0.01	1.20	1.03, 1.40	0.02
Demographics									
Age (years)	1.00	0.99, 1.00	0.26	1.00	0.99, 1.00	0.92	1.00	0.99, 1.00	0.17
Female	1.40	1.30, 1.50	<0.01	1.48	1.34, 1.63	<0.01	1.36	1.24, 1.50	<0.01
Married	0.95	0.89, 1.02	0.14	0.95	0.86, 1.04	0.29	0.94	0.85, 1.03	0.20
Specialist care diagnosis									
CCI	1.02	0.94, 1.10	0.62	1.05	0.93, 1.19	0.41	1.02	0.92, 1.14	0.71
Psoriasis	0.87	0.81, 0.93	<0.01	0.97	0.88, 1.08	0.59	0.79	0.71, 0.87	<0.01
Crohn’s disease	0.81	0.63, 1.04	0.10	0.82	0.57, 1.17	0.27	0.82	0.57, 1.16	0.26
RA	1.01	0.88, 1.15	0.92	0.93	0.77, 1.13	0.46	1.08	0.90, 1.29	0.43
Ulcerative colitis	0.96	0.74, 1.26	0.79	0.78	0.54, 1.12	0.18	1.33	0.96, 1.84	0.09
Axial involvement	1.20	1.01, 1.44	0.04	1.24	0.98, 1.57	0.07	1.16	0.88, 1.53	0.29
Type 2 diabetes	0.99	0.85, 1.16	0.89	0.90	0.70, 1.17	0.43	1.04	0.86, 1.26	0.69
Uveitis	0.88	0.70, 1.11	0.29	1.07	0.79, 1.46	0.66	0.82	0.60, 1.13	0.23
Biologic treatment initiation									
Time since disease onset (years)	0.99	0.98, 1.00	0.01	0.98	0.97, 1.00	0.01	0.99	0.98, 1.01	0.32
1 prior line biologic experience (*vs* naïve)	1.37	1.26, 1.48	<0.01						
2 prior lines biologic experience (*vs* naïve)	1.53	1.35, 1.75	<0.01						
≥3 prior lines biologic experience (*vs* naïve)	1.78	1.52, 2.09	<0.01						

Sensitivity analyses implementing a fixed 90 day grace period revealed that time to non-persistence was sensitive to the definition of the grace period. In this analysis, risk of non-persistence from both UST and SEC was significantly lower than for ADA (see Supplementary Tables S5 and S6, available at *Rheumatology Advances in Practice* online), as in the main analysis.

## Discussion

Treatment persistence is a composite measure of effectiveness, safety, tolerability and general patient satisfaction with biologic treatment. In this study we provide the first results comparing multiple IL inhibitor treatments against TNFi treatment in Swedish PsA patients. Overall, in the primary analysis, real-world PsA patients treated with UST exhibited favourable persistence profiles compared with ADA and SEC, independently if used in biologic-naïve or biologic-experienced patients. The performance of SEC varied based on biologic experience, where it was favourable relative to ADA in biologic-naïve patients but unfavourable in biologic-experienced patients. Relative persistence with SEC was sensitive to the assumed secukinumab rate of consumption, as well as the definition of the grace period. Given the many biologic treatment options available in today’s modern treatment environment, the findings from the present study may be used to help physicians optimize patients’ treatment pathways. Societal resources used to pay for medications and other related cost may be more efficiently allocated by avoiding treatments with a higher risk of failure, in addition to avoiding unnecessary patient and healthcare burdens associated with treatment non-persistence and switching.

The Kaplan–Meier data, representing a period of up to 10.6 years, showed that patients treated with UST, SEC and ADA, regardless of their characteristics, often discontinue at the lowest rates with UST. The overall results were in line with previous findings, in which UST had the highest persistence rate in the studied biologics throughout the whole follow-up period relative to ADA [22, 25]. The findings were also consistent with research studying psoriasis patients, where UST was associated with the highest persistence rate [32–43].

Statistically significant risk factors of non-persistence in PsA patients were found to be ADA use, female sex, axial involvement, recent disease onset and increasing experience with biologics. Psoriasis diagnosed in specialist care was associated with a lower risk of non-persistence, except in biologic-naïve patients. Other variations were observed when examining biologic experience subgroups: ulcerative colitis was associated with a higher risk of non-persistence in biologic-experienced patients only, and time since disease onset did not appear to be a risk factor for biologic-experienced patients. However, as the HR estimate of time since disease onset was close to 1 overall and in the biologic-experienced subgroup, the clinical importance is unclear. Recent studies showed that biologic-naïve PsA patients were more persistent than biologic-experienced patients [8–10, 17, 19], which was also observed in some studies of psoriasis patients [34, 44]. Some existing literature reports that time from disease onset is associated with non-persistence, as in the present study [8, 10, 11], although the association magnitude (1–2% risk reduction per year) appears to be small and even non-significant in the case of biologic-experienced patients. The present study found that women have a higher risk of non-persistence, consistent with most other findings [10–12, 14, 16, 17, 19, 20].

Most previous research included a narrow study population, excluding patients with a recorded diagnosis code in specialist care for axial involvement, Crohn’s disease, RA or ulcerative colitis [8, 9, 39, 45]. The present study includes a wide patient population, electing to include all relevant PsA patients and adjusting for, instead of excluding, previously recorded diagnoses of psoriasis, Crohn’s disease, RA, ulcerative colitis, axial involvement, type 2 diabetes and uveitis. This allows for an analysis of a full patient population while investigating the effect prior records of various diagnoses have on persistence, reflecting a comprehensive real-world PsA patient group. Patients with PsA in the present study receiving ustekinumab more often had a prior diagnosis of psoriasis. Ustekinumab is known to be effective against psoriasis, which may contribute to improved persistence [32–43]. All patients in the present study have a primary diagnosis of PsA, indicating that they suffer from joint involvement. However, those with comorbid skin psoriasis may be more likely to be persistent if their skin is sufficiently treated, even when PsA is not optimally treated.

The administrative registry data used in this study has many advantages but also some challenges in the study of treatment non-persistence including the identification of treatment switching, volume of drug consumed and definition of the grace period. Treatment switching to another biologic is an identifiable event for all biologics except hospital-procured products, which are not always included in the PDR. The incomplete recording contributes to misclassification of patients between biologic-naïve and experienced subgroups and some unobserved treatment switching. The duration of drug supply required assumptions about the volume of drug consumed by the patient, as neither physician instructions (dosing instructions), patient weight (relevant for dosing) or patient behaviour (actual consumption) is available in the data. Patients are likely to consume the indicated dose according to treatment posology in many cases, which was assumed in this study. However, bias due to dose escalation, non-adherence and similar phenomena may be present.

The grace period in the present study’s main analysis was assumed to be dynamic, equal to the supplied days of drug at each dispensation. This incorporates patient behaviour through the act of dispensation from the pharmacy, thereby reflecting real-world patient behaviour. A dynamic grace period has the advantage of accounting for variations in patient consumption that are proportional to the amount of drug dispensed. A disadvantage is that it is dependent on each treatment’s posology, as treatments dispensed less frequently will have longer grace periods by definition. Ustekinumab’s posology may contribute to the high persistence rates observed, as the infrequent dosing schedule, relative to other treatments, places a low administrative burden on patients. Fixed grace periods are often used in persistence studies [23, 24, 34, 39, 46] and were therefore also analysed in the present study. The results of the proportional hazards model using a fixed grace period compared with a dynamic one differed both comparatively and in magnitude. Using a fixed grace period in the overall group, absolute persistence rates were very similar for ADA, UST and SEC while the adjusted results showed that persistence on UST and SEC were similar, and both were better than ADA.

PsA treatment guidelines [47] often recommend TNFis over IL inhibitors as a first-line biologic. This explains why ADA is much more prevalent in biologic-naïve patients (91%). The high proportion of first-line ADA patients reflects general clinical practice and has been seen on a European level [21]. This was accounted for through the adjustment for treatment line. Adding to the difference in patient numbers between exposure groups is the date of market authorization, where, for the PsA indication under study, ADA was available first, followed by UST and SEC.

The present study included two IL inhibitor medications (UST and SEC) with a reasonable follow-up duration. Other biologics targeting similar IL inhibition pathways as those included in the present study, such as ixekizumab, brodalumab and guselkumab, should be assessed when the PsA indication is approved and sufficient follow-up data become available, as should treatments along other pathways, including JAK inhibitors. Adalimumab is a common treatment in Sweden and throughout Europe and is, as such, relevant as a comparator. Although a relevant alternative TNFi therapy, etanercept was not included in this study, which is a limitation. Future research should consider assessing etanercept to identify settings where different TNFis may diverge in persistence.

The study uses Swedish data and the generalizability of the results may therefore be limited to settings that have similar clinical practices, available treatments and patient characteristics as the Swedish PsA population. Persistence levels can vary between studies using administrative data and clinical registries, which has led to discussions about how results derived from these types of data should be interpreted [48]. There seem to be systematic differences in persistence estimates derived from these two data sources. Future studies may explore these differences in order to provide valid comparisons between persistence measurements calculated using different data sources.

## Conclusions

Treatment persistence represents overall treatment effectiveness, safety, tolerability and patient satisfaction for those treated with biologics in PsA. Ustekinumab exhibits a generally favourable treatment persistency profile in both biologic-naïve and experienced patients, while SEC exhibits favourable persistency in biologic-naïve but not in biologic-experienced patients compared with ADA. Aside from medication, risk factors for reduced treatment persistence include female sex, axial involvement, recent disease onset and increasing biologic treatment experience. Psoriasis was associated with a lower risk of non-persistence overall and in biologic-experienced patients. Treatment persistency and other risk factors should be considered in clinical practice to determine an optimal treatment plan for patients. Improved treatment planning leading to higher persistence rates directly contributes to reduced patient burden and efficient allocation of societal economic resources.

## Supplementary Material

rkaa070_Supplementary_Data
