# Corrigendum to: Persistence of biologic treatments in psoriatic arthritis: a population-based study in Sweden

**DOI:** 10.1093/rap/rkab005

**Published:** 2021-02-12

**Authors:** Kirk Geale, Ingrid Lindberg, Emma C Paulsson, E Christina M Wennerström, Anna Tjärnlund, Wim Noel, Dana Enkusson, Elke Theander

**Affiliations:** 1 Quantify Research, Stockholm; 2 Department of Public Health and Clinical Medicine, Umeå University, Umeå; 3 Janssen-Cilag AB, Solna, Sweden; 4 Department of Epidemiology Research, Statens Serum Institut, Copenhagen, Denmark; 5 Janssen Pharmaceutica NV, Beerse, Belgium

Rheumatology Advances in Practice 2020. doi:10.1093/rap/rkaa070

In the originally published version of this manuscript, an error was noted in the Conclusions section. The following sentence should read: “Ustekinumab exhibits a generally favourable treatment persistency profile in both biologic-naïve and experienced patients, while SEC exhibits favourable persistency in biologic-naive but not in biologic-experienced patients compared with ADA” instead of “Ustekinumab exhibits a generally favourable treatment persistency profile in both biologic-naïve and experienced patients, while SEC exhibits favourable persistency in biologic-experienced but not in biologic-naïve patients compared with ADA”.

This error has now been corrected online.

