# Supplementary material for: Persistence of biologic treatments in psoriatic arthritis: a population-based study in Sweden
Source: Rheumatol Adv Pract. 2020 Dec 19;4(2):rkaa070. doi: 10.1093/rap/rkaa070 (PMC7772250; doi:10.1093/rap/rkaa070)
Supplement: rkaa070_Supplementary_Data [file rkaa070_supplementary_data.docx]

**Supplementary Table S1: ICD-10 codes for included comorbidities**

| **Comorbidity/Diagnosis** | **ICD-10 code** |
| --- | --- |
| Psoriasis | L40.0-4 or L40.8 or L40.9 |
| Crohn’s disease | K50+ |
| Rheumatoid arthritis | M05+ or M06+ |
| Ulcerative colitis | K51+ |
| Axial involvement | M45+ or M47+ |
| Uveitis | H20+ |
| Diabetes type 2 | E11+ |

***** *ICD-10 = The International Classification of Diseases, 10th Revision.*

**Supplementary Table S2: Charlson Comorbidity Index Code List**

| **Comorbidity** | **ICD-10 diagnosis codes** | **Weight** |
| --- | --- | --- |
| Myocardial infarction | I21+, I22+, I25.2 | 0 |
| Congestive heart failure | I09.9, I11.0, I13.0, I13.2, I25.5, I42.0, I42.5–I42.9, I43+, I50+, P29.0 | 2 |
| Peripheral vascular disease | I70+, I71+, I73.1, I73.8, I73.9, I77.1, I79.0, I79.2, K55.1, K55.8, K55.9, Z95.8, Z95.9 | 0 |
| Cerebrovascular disease | G45+, G46+, H34.0, I60+ –I69+ | 0 |
| Dementia | F00.x–F03.x, F05.1, G30.x, G31.1 | 2 |
| Chronic pulmonary disease | I27.8, I27.9, J40.x–J47.x, J60.x–J67.x, J68.4, J70.1, J70.3 | 1 |
| Rheumatologic disease | M05.x, M06.x, M31.5, M32.x–M34.x, M35.1, M35.3, M36.0 | 1 |
| Peptic ulcer disease | K25.x–K28.x | 0 |
| Mild liver disease* | B18.x, K70.0–K70.3, K70.9, K71.3–K71.5, K71.7, K73.x, K74.x, K76.0, K76.2–K76.4, K76.8, K76.9, Z94.4 | 2 |
| Diabetes without chronic complications* | E10.0, E10.1, E10.6, E10.8, E10.9, E11.0, E11.1, E11.6, E11.8, E11.9, E12.0, E12.1, E12.6, E12.8, E12.9, E13.0, E13.1, E13.6, E13.8, E13.9, E14.0, E14.1, E14.6, E14.8, E14.9 | 0 |
| Diabetes with chronic complications* | E10.2–E10.5, E10.7, E11.2–E11.5, E11.7, E12.2–E12.5, E12.7, E13.2– E13.5, E13.7, E14.2–E14.5, E14.7 | 1 |
| Hemiplegia or paraplegia | G04.1, G11.4, G80.1, G80.2, G81.x, G82.x, G83.0–G83.4, G83.9 | 2 |
| Renal disease | I12.0, I13.1, N03.2–N03.7, N05.2– N05.7, N18.x, N19.x, N25.0, Z49.0– Z49.2, Z94.0, Z99.2 | 1 |
| Any malignancy, including leukemia and lymphoma* | C00.x–C26.x, C30.x–C34.x, C37.x– C41.x, C43.x, C45.x–C58.x, C60.x– C76.x, C81.x–C85.x, C88.x, C90.x–C97.x | 2 |
| Moderate to severe liver disease* | I85.0, I85.9, I86.4, I98.2, K70.4, K71.1, K72.1, K72.9, K76.5, K76.6, K76.7 | 4 |
| Metastatic solid tumor* | C77.x–C80.x | 6 |
| AIDS/HIV | B20.x–B22.x, B24.x | 4 |
| Maximum comorbidity score |  | 24 |

*Source: ^1^. Abbreviations: AIDS = Acquired Immunodeficiency Syndrome, HIV = Human Immunodeficiency Virus, ICD-10 = The International Classification of Diseases, 10th Revision. *The following comorbidities are mutually exclusive: diabetes with and without chronic complications; mild and moderate to severe liver disease; and any malignancy and metastatic solid tumor.*

1. Quan H, Li B, Couris CM, et al. Updating and validating the Charlson comorbidity index and score for risk adjustment in hospital discharge abstracts using data from 6 countries. *American journal of epidemiology* 2011;173(6):676-82.

**Supplementary Table S3: Results from unadjusted Cox proportional hazards model – Time to treatment non-persistence**

|  | **Overall (N= 4,649)** | | | **Biologic naïve (N= 2,177)** | | | **Biologic experienced (N= 2,472)** | | |
| --- | --- | --- | --- | --- | --- | --- | --- | --- | --- |
|  | **HR** | **95% CI** | **p-value** | **HR** | **95% CI** | **p-value** | **HR** | **95% CI** | **p-value** |
| **Treatment (vs adalimumab)** |  |  |  |  |  |  |  |  |  |
| Ustekinumab | 0.67 | 0.6 - 0.75 | <0.01 | 0.48 | 0.34 - 0.69 | <0.01 | 0.60 | 0.53 - 0.68 | <0.01 |
| Secukinumab | 1.12 | 1.03 - 1.23 | 0.01 | 0.55 | 0.43 - 0.71 | <0.01 | 1.08 | 0.97 - 1.2 | 0.18 |

**Supplementary Table S4: Results from adjusted Cox proportional hazards model – Time to treatment non-persistence**

|  | **Overall (N= 4,649)** | | | **Biologic naïve (N= 2,177)** | | | **Biologic experienced (N= 2,472)** | | |
| --- | --- | --- | --- | --- | --- | --- | --- | --- | --- |
|  | **HR** | **95% CI** | **p-value** | **HR** | **95% CI** | **p-value** | **HR** | **95% CI** | **p-value** |
| **Treatment (vs adalimumab)** |  |  |  |  |  |  |  |  |  |
| Ustekinumab | 0.56 | 0.49 - 0.64 | <0.01 | 0.48 | 0.33 - 0.69 | <0.01 | 0.65 | 0.56 - 0.76 | <0.01 |
| Secukinumab | 1.01 | 0.88 - 1.15 | 0.91 | 0.65 | 0.49 - 0.86 | <0.01 | 1.20 | 1.03 - 1.40 | 0.02 |
| **Demographics** |  |  |  |  |  |  |  |  |  |
| Age (years) | 1.00 | 0.99 - 1.00 | 0.26 | 1.00 | 0.99 - 1.00 | 0.92 | 1.00 | 0.99 - 1.00 | 0.17 |
| Female | 1.40 | 1.30 - 1.50 | <0.01 | 1.48 | 1.34 - 1.63 | <0.01 | 1.36 | 1.24 - 1.50 | <0.01 |
| Married | 0.95 | 0.89 - 1.02 | 0.14 | 0.95 | 0.86 - 1.04 | 0.29 | 0.94 | 0.85 - 1.03 | 0.20 |
| **Specialist care diagnosis (Y/N)** |  |  |  |  |  |  |  |  |  |
| Charlson Comorbidity Index | 1.02 | 0.94 - 1.10 | 0.62 | 1.05 | 0.93 - 1.19 | 0.41 | 1.02 | 0.92 - 1.14 | 0.71 |
| Psoriasis | 0.87 | 0.81 - 0.93 | <0.01 | 0.97 | 0.88 - 1.08 | 0.59 | 0.79 | 0.71 - 0.87 | <0.01 |
| Crohn’s disease | 0.81 | 0.63 - 1.04 | 0.10 | 0.82 | 0.57 - 1.17 | 0.27 | 0.82 | 0.57 - 1.16 | 0.26 |
| Rheumatoid arthritis | 1.01 | 0.88 - 1.15 | 0.92 | 0.93 | 0.77 - 1.13 | 0.46 | 1.08 | 0.90 - 1.29 | 0.43 |
| Ulcerative colitis | 0.96 | 0.74 - 1.26 | 0.79 | 0.78 | 0.54 - 1.12 | 0.18 | 1.33 | 0.96 - 1.84 | 0.09 |
| Axial involvement | 1.20 | 1.01 - 1.44 | 0.04 | 1.24 | 0.98 - 1.57 | 0.07 | 1.16 | 0.88 - 1.53 | 0.29 |
| Diabetes type 2 | 0.99 | 0.85 - 1.16 | 0.89 | 0.90 | 0.70 - 1.17 | 0.43 | 1.04 | 0.86 - 1.26 | 0.69 |
| Uveitis | 0.88 | 0.70 - 1.11 | 0.29 | 1.07 | 0.79 - 1.46 | 0.66 | 0.82 | 0.60 - 1.13 | 0.23 |
| **Treatment initiation** |  |  |  |  |  |  |  |  |  |
| Time since disease onset (years) | 0.99 | 0.98 - 1.00 | 0.01 | 0.98 | 0.97 - 1.00 | 0.01 | 0.99 | 0.98 - 1.01 | 0.32 |
| 1 prior line of biologic experience (vs naïve) | 1.37 | 1.26 - 1.48 | <0.01 |  |  |  |  |  |  |
| 2 prior lines of biologic experience (vs naïve) | 1.53 | 1.35 - 1.75 | <0.01 |  |  |  |  |  |  |
| 3+ prior lines of biologic experience (vs naïve) | 1.78 | 1.52 - 2.09 | <0.01 |  |  |  |  |  |  |
| **Index year (vs 2008)** |  |  |  |  |  |  |  |  |  |
| Index year 2009 | 0.93 | 0.79 - 1.09 | 0.37 | 0.88 | 0.71 - 1.07 | 0.20 | 1.07 | 0.82 - 1.40 | 0.62 |
| Index year 2010 | 1.07 | 0.90 - 1.27 | 0.44 | 1.04 | 0.85 - 1.28 | 0.71 | 1.15 | 0.86 - 1.53 | 0.35 |
| Index year 2011 | 1.02 | 0.86 - 1.20 | 0.82 | 0.97 | 0.79 - 1.18 | 0.75 | 1.10 | 0.81 - 1.49 | 0.54 |
| Index year 2012 | 1.22 | 1.03 - 1.43 | 0.02 | 1.21 | 1.00 - 1.47 | 0.05 | 1.24 | 0.94 - 1.63 | 0.12 |
| Index year 2013 | 1.12 | 0.95 - 1.33 | 0.16 | 1.16 | 0.94 - 1.44 | 0.15 | 1.05 | 0.81 - 1.37 | 0.70 |
| Index year 2014 | 1.02 | 0.86 - 1.20 | 0.83 | 0.98 | 0.79 - 1.22 | 0.88 | 1.06 | 0.82 - 1.37 | 0.68 |
| Index year 2015 | 1.10 | 0.93 - 1.30 | 0.26 | 1.26 | 1.02 - 1.56 | 0.03 | 0.97 | 0.75 - 1.26 | 0.83 |
| Index year 2016 | 0.91 | 0.76 - 1.07 | 0.25 | 0.83 | 0.65 - 1.06 | 0.14 | 0.95 | 0.74 - 1.23 | 0.70 |
| Index year 2017 | 1.01 | 0.85 - 1.20 | 0.95 | 0.96 | 0.74 - 1.24 | 0.76 | 1.02 | 0.79 - 1.32 | 0.87 |
| Index year 2018 | 0.86 | 0.69 - 1.09 | 0.21 | 0.70 | 0.43 - 1.11 | 0.13 | 0.89 | 0.66 - 1.21 | 0.46 |
| **County of residence (vs Blekinge county)** |  |  |  |  |  |  |  |  |  |
| Dalarna county | 0.96 | 0.72 - 1.27 | 0.76 | 0.74 | 0.46 - 1.17 | 0.20 | 1.18 | 0.80 - 1.73 | 0.40 |
| Gavleborg county | 0.69 | 0.53 - 0.91 | 0.01 | 0.63 | 0.39 - 1.00 | 0.05 | 0.69 | 0.49 - 0.96 | 0.03 |
| Gotland county | 0.91 | 0.66 - 1.25 | 0.56 | 0.78 | 0.46 - 1.30 | 0.34 | 1.07 | 0.69 - 1.65 | 0.78 |
| Halland county | 0.83 | 0.64 - 1.07 | 0.15 | 0.94 | 0.63 - 1.40 | 0.74 | 0.68 | 0.47 - 0.98 | 0.04 |
| Jamtland county | 1.12 | 0.86 - 1.47 | 0.39 | 1.09 | 0.72 - 1.64 | 0.69 | 1.19 | 0.81 - 1.73 | 0.38 |
| Jonkoping county | 0.75 | 0.55 - 1.03 | 0.08 | 0.84 | 0.54 - 1.30 | 0.44 | 0.74 | 0.48 - 1.15 | 0.18 |
| Kalmar county | 0.82 | 0.63 - 1.05 | 0.11 | 0.83 | 0.54 - 1.26 | 0.38 | 0.77 | 0.55 - 1.07 | 0.12 |
| Kronoberg county | 0.80 | 0.59 - 1.08 | 0.15 | 1.12 | 0.70 - 1.79 | 0.63 | 0.52 | 0.34 - 0.80 | <0.01 |
| Norrbotten county | 0.94 | 0.72 - 1.22 | 0.63 | 0.99 | 0.66 - 1.50 | 0.98 | 0.85 | 0.59 - 1.23 | 0.40 |
| Orebro county | 0.78 | 0.60 - 1.03 | 0.08 | 0.74 | 0.48 - 1.15 | 0.18 | 0.80 | 0.56 - 1.15 | 0.23 |
| Ostergotland | 0.62 | 0.47 - 0.81 | <0.01 | 0.62 | 0.38 - 0.99 | 0.05 | 0.58 | 0.41 - 0.82 | <0.01 |
| Skane county | 0.85 | 0.68 - 1.06 | 0.16 | 0.86 | 0.59 - 1.25 | 0.43 | 0.81 | 0.60 - 1.09 | 0.16 |
| Sodermanland county | 0.63 | 0.47 - 0.83 | <0.01 | 0.56 | 0.34 - 0.95 | 0.03 | 0.64 | 0.46 - 0.88 | 0.01 |
| Stockholm county | 0.78 | 0.63 - 0.96 | 0.02 | 0.83 | 0.58 - 1.19 | 0.31 | 0.73 | 0.56 - 0.97 | 0.03 |
| Uppsala county | 0.69 | 0.53 - 0.88 | <0.01 | 0.62 | 0.40 - 0.94 | 0.02 | 0.73 | 0.53 - 1.02 | 0.06 |
| Varmland county | 0.88 | 0.67 - 1.16 | 0.37 | 0.79 | 0.52 - 1.21 | 0.27 | 0.99 | 0.67 - 1.45 | 0.94 |
| Vasterbotten county | 0.96 | 0.71 - 1.28 | 0.76 | 1.09 | 0.69 - 1.69 | 0.72 | 0.87 | 0.59 - 1.29 | 0.49 |
| Vastermanland county | 0.82 | 0.62 - 1.07 | 0.14 | 0.82 | 0.54 - 1.24 | 0.34 | 0.80 | 0.55 - 1.17 | 0.26 |
| Vasternorrland county | 0.73 | 0.57 - 0.94 | 0.01 | 0.76 | 0.51 - 1.11 | 0.16 | 0.63 | 0.43 - 0.92 | 0.02 |
| Vastra Gotaland county | 0.65 | 0.52 - 0.82 | <0.01 | 0.71 | 0.49 - 1.04 | 0.08 | 0.59 | 0.44 - 0.80 | <0.01 |

*Note: Y/N = Yes/No.*

**Supplementary Table S5: Cox proportional hazards model of time to non-persistence with biologics in PsA treatment exposures, fixed 90-day grace period, unadjusted model**

|  | **Overall (N= 4,649)** | | | **Biologic naïve (N= 2,177)** | | | **Biologic experienced (N= 2,472)** | | |
| --- | --- | --- | --- | --- | --- | --- | --- | --- | --- |
|  | **HR** | **95% CI** | **p-value** | **HR** | **95% CI** | **p-value** | **HR** | **95% CI** | **p-value** |
| **Treatment (vs adalimumab)** |  |  |  |  |  |  |  |  |  |
| Ustekinumab | 1.00 | 0.89 - 1.12 | 0.98 | 0.77 | 0.55 - 1.09 | 0.15 | 0.85 | 0.75 - 0.96 | 0.01 |
| Secukinumab | 1.02 | 0.93 - 1.13 | 0.64 | 0.84 | 0.65 - 1.08 | 0.17 | 0.86 | 0.76 - 0.97 | 0.01 |

**Supplementary Table S6: Cox proportional hazards model of time to non-persistence with biologics in PsA treatment exposures, fixed 90-day grace period, adjusted model**

|  | **Overall (N= 4,649)** | | | **Biologic naïve (N= 2,177)** | | | **Biologic experienced (N= 2,472)** | | |
| --- | --- | --- | --- | --- | --- | --- | --- | --- | --- |
|  | **HR** | **95% CI** | **p-value** | **HR** | **95% CI** | **p-value** | **HR** | **95% CI** | **p-value** |
| **Treatment (vs adalimumab)** |  |  |  |  |  |  |  |  |  |
| Ustekinumab | 0.81 | 0.70 - 0.94 | <0.01 | 0.76 | 0.53 - 1.11 | 0.15 | 0.92 | 0.79 - 1.07 | 0.28 |
| Secukinumab | 0.82 | 0.70 - 0.95 | 0.01 | 0.91 | 0.67 - 1.22 | 0.53 | 0.90 | 0.76 - 1.07 | 0.23 |
| **Demographics** |  |  |  |  |  |  |  |  |  |
| Age (years) | 1.00 | 0.99 - 1.00 | 0.27 | 1.00 | 1.00 - 1.00 | 0.90 | 1.00 | 0.99 - 1.00 | 0.07 |
| Female | 1.41 | 1.31 - 1.52 | <0.01 | 1.58 | 1.43 - 1.76 | <0.01 | 1.29 | 1.16 - 1.44 | <0.01 |
| Married | 0.96 | 0.89 - 1.03 | 0.29 | 0.91 | 0.82 - 1.01 | 0.08 | 1.01 | 0.91 - 1.12 | 0.86 |
| **Specialist care diagnosis (Y/N)** |  |  |  |  |  |  |  |  |  |
| Charlson Comorbidity Index | 1.06 | 0.98 - 1.15 | 0.15 | 1.05 | 0.91 - 1.20 | 0.51 | 1.06 | 0.95 - 1.18 | 0.32 |
| Psoriasis | 0.87 | 0.81 - 0.94 | <0.01 | 0.97 | 0.87 - 1.08 | 0.56 | 0.79 | 0.71 - 0.88 | <0.01 |
| Crohn’s disease | 0.75 | 0.57 - 0.99 | 0.04 | 0.73 | 0.50 - 1.07 | 0.11 | 0.81 | 0.56 - 1.17 | 0.26 |
| Rheumatoid arthritis | 1.00 | 0.87 - 1.14 | 0.96 | 0.93 | 0.75 - 1.16 | 0.51 | 1.07 | 0.89 - 1.29 | 0.47 |
| Ulcerative colitis | 1.00 | 0.76 - 1.32 | 0.98 | 0.87 | 0.60 - 1.25 | 0.45 | 1.24 | 0.83 - 1.87 | 0.29 |
| Axial involvement | 1.24 | 1.03 - 1.50 | 0.03 | 1.30 | 1.00 - 1.70 | 0.05 | 1.17 | 0.89 - 1.54 | 0.27 |
| Diabetes type 2 | 1.00 | 0.85 - 1.18 | 0.98 | 0.93 | 0.69 - 1.25 | 0.63 | 1.06 | 0.87 - 1.28 | 0.58 |
| Uveitis | 0.76 | 0.59 - 0.99 | 0.04 | 0.91 | 0.60 - 1.37 | 0.65 | 0.70 | 0.50 - 0.97 | 0.03 |
| **Treatment initiation** |  |  |  |  |  |  |  |  |  |
| Time since disease onset (years) | 0.99 | 0.98 - 1.00 | 0.01 | 0.98 | 0.96 - 0.99 | <0.01 | 0.99 | 0.98 - 1.01 | 0.41 |
| 1 prior line of biologic experience (vs naïve) | 1.36 | 1.24 - 1.48 | <0.01 |  |  |  |  |  |  |
| 2 prior lines of biologic experience (vs naïve) | 1.58 | 1.37 - 1.83 | <0.01 |  |  |  |  |  |  |
| 3+ prior lines of biologic experience (vs naïve) | 1.76 | 1.48 - 2.10 | <0.01 |  |  |  |  |  |  |
| **Index year (vs 2008)** |  |  |  |  |  |  |  |  |  |
| Index year 2009 | 0.97 | 0.81 - 1.15 | 0.71 | 0.99 | 0.79 - 1.23 | 0.91 | 0.96 | 0.72 - 1.27 | 0.75 |
| Index year 2010 | 1.11 | 0.93 - 1.32 | 0.26 | 1.15 | 0.93 - 1.44 | 0.20 | 1.05 | 0.78 - 1.41 | 0.74 |
| Index year 2011 | 1.07 | 0.89 - 1.27 | 0.48 | 1.02 | 0.82 - 1.27 | 0.87 | 1.17 | 0.86 - 1.58 | 0.31 |
| Index year 2012 | 1.19 | 1.00 - 1.42 | 0.05 | 1.27 | 1.03 - 1.58 | 0.03 | 1.10 | 0.82 - 1.48 | 0.52 |
| Index year 2013 | 1.14 | 0.95 - 1.35 | 0.16 | 1.23 | 0.99 - 1.54 | 0.07 | 1.00 | 0.75 - 1.33 | 0.99 |
| Index year 2014 | 1.02 | 0.85 - 1.22 | 0.82 | 1.08 | 0.84 - 1.39 | 0.54 | 0.95 | 0.73 - 1.25 | 0.74 |
| Index year 2015 | 1.18 | 0.98 - 1.41 | 0.08 | 1.36 | 1.07 - 1.73 | 0.01 | 0.99 | 0.76 - 1.3 | 0.96 |
| Index year 2016 | 1.06 | 0.88 - 1.27 | 0.54 | 1.00 | 0.77 - 1.31 | 0.98 | 1.03 | 0.79 - 1.34 | 0.83 |
| Index year 2017 | 1.07 | 0.88 - 1.29 | 0.51 | 1.09 | 0.81 - 1.47 | 0.55 | 0.98 | 0.75 - 1.29 | 0.90 |
| Index year 2018 | 0.75 | 0.57 - 1.00 | 0.05 | 0.78 | 0.44 - 1.39 | 0.40 | 0.67 | 0.47 - 0.96 | 0.03 |
| **County of residence (vs Blekinge county)** |  |  |  |  |  |  |  |  |  |
| Dalarna county | 0.78 | 0.59 - 1.05 | 0.10 | 0.54 | 0.33 - 0.88 | 0.01 | 0.96 | 0.66 - 1.41 | 0.84 |
| Gavleborg county | 0.63 | 0.48 - 0.83 | <0.01 | 0.46 | 0.27 - 0.77 | <0.01 | 0.71 | 0.51 - 0.99 | 0.04 |
| Gotland county | 0.76 | 0.53 - 1.08 | 0.13 | 0.56 | 0.30 - 1.04 | 0.07 | 0.94 | 0.61 - 1.43 | 0.77 |
| Halland county | 0.64 | 0.49 - 0.84 | <0.01 | 0.65 | 0.42 - 1.01 | 0.06 | 0.56 | 0.37 - 0.82 | <0.01 |
| Jamtland county | 0.80 | 0.61 - 1.05 | 0.11 | 0.79 | 0.51 - 1.23 | 0.30 | 0.72 | 0.50 - 1.06 | 0.10 |
| Jonkoping county | 0.53 | 0.38 - 0.73 | <0.01 | 0.39 | 0.24 - 0.63 | <0.01 | 0.68 | 0.44 - 1.06 | 0.09 |
| Kalmar county | 0.71 | 0.54 - 0.93 | 0.01 | 0.54 | 0.34 - 0.86 | 0.01 | 0.83 | 0.59 - 1.16 | 0.27 |
| Kronoberg county | 0.62 | 0.46 - 0.84 | <0.01 | 0.63 | 0.39 - 1.02 | 0.06 | 0.54 | 0.36 - 0.82 | <0.01 |
| Norrbotten county | 0.74 | 0.57 - 0.96 | 0.02 | 0.67 | 0.43 - 1.04 | 0.07 | 0.72 | 0.50 - 1.04 | 0.08 |
| Orebro county | 0.61 | 0.47 - 0.81 | <0.01 | 0.51 | 0.33 - 0.80 | <0.01 | 0.69 | 0.48 - 0.99 | 0.04 |
| Ostergotland | 0.56 | 0.43 - 0.74 | <0.01 | 0.54 | 0.34 - 0.87 | 0.01 | 0.55 | 0.39 - 0.78 | <0.01 |
| Skane county | 0.69 | 0.55 - 0.87 | <0.01 | 0.59 | 0.39 - 0.89 | 0.01 | 0.72 | 0.53 - 0.97 | 0.03 |
| Sodermanland county | 0.59 | 0.44 - 0.79 | <0.01 | 0.51 | 0.30 - 0.87 | 0.01 | 0.62 | 0.43 - 0.88 | 0.01 |
| Stockholm county | 0.66 | 0.53 - 0.81 | <0.01 | 0.56 | 0.38 - 0.82 | <0.01 | 0.72 | 0.55 - 0.94 | 0.02 |
| Uppsala county | 0.63 | 0.49 - 0.81 | <0.01 | 0.55 | 0.35 - 0.85 | 0.01 | 0.66 | 0.47 - 0.93 | 0.02 |
| Varmland county | 0.61 | 0.46 - 0.82 | <0.01 | 0.51 | 0.33 - 0.81 | <0.01 | 0.65 | 0.43 - 1.00 | 0.05 |
| Vasterbotten county | 0.62 | 0.45 - 0.85 | <0.01 | 0.69 | 0.43 - 1.13 | 0.14 | 0.54 | 0.35 - 0.82 | <0.01 |
| Vastermanland county | 0.64 | 0.49 - 0.85 | <0.01 | 0.52 | 0.34 - 0.81 | <0.01 | 0.74 | 0.50 - 1.11 | 0.14 |
| Vasternorrland county | 0.63 | 0.48 - 0.81 | <0.01 | 0.53 | 0.35 - 0.81 | <0.01 | 0.67 | 0.46 - 0.99 | 0.05 |
| Vastra Gotaland county | 0.60 | 0.48 - 0.76 | <0.01 | 0.61 | 0.41 - 0.92 | 0.02 | 0.56 | 0.41 - 0.76 | <0.01 |

*Note: Y/N = Yes/No*
